# The impact of stratified immunity on the transmission dynamics of influenza

**DOI:** 10.1016/j.epidem.2017.03.003

**Published:** 2017-09

**Authors:** Hsiang-Yu Yuan, Marc Baguelin, Kin O. Kwok, Nimalan Arinaminpathy, Edwin van Leeuwen, Steven Riley

**Affiliations:** a MRC Centre for Outbreak Analysis and Disease Modelling, Department of Infectious Disease Epidemiology, School of Public Health, Imperial College London, London, United Kingdom; b Respiratory Diseases Department, Public Health England, London, United Kingdom; c Centre for the Mathematical Modelling of Infectious Disease, Department of Infectious Disease Epidemiology, London School of Hygiene & Tropical Medicine, London, United Kingdom; d The Jockey Club School of Public Health and Primary Care, The Chinese University of Hong Kong, Hong Kong Special Administrative Region, China; e Stanley Ho Centre for Emerging Infectious Diseases, The Chinese University of Hong Kong, Hong Kong Special Administrative Region, China; f WHO Collaborating Centre for Infectious Disease Epidemiology and Control, School of Public Health, Li Ka Shing Faculty of Medicine, The University of Hong Kong, Hong Kong Special Administrative Region, China

**Keywords:** Influenza, Epidemic model, Stratified immunity, Antibody responses, Age-specific seroprevalence, Inferring transmission dynamics

## Abstract

Although empirical studies show that protection against influenza infection in humans is closely related to antibody titres, influenza epidemics are often described under the assumption that individuals are either susceptible or not. Here we develop a model in which antibody titre classes are enumerated explicitly and mapped onto a variable scale of susceptibility in different age groups. Fitting only with pre- and post-wave serological data during 2009 pandemic in Hong Kong, we demonstrate that with stratified immunity, the timing and the magnitude of the epidemic dynamics can be reconstructed more accurately than is possible with binary seropositivity data. We also show that increased infectiousness of children relative to adults and age-specific mixing are required to reproduce age-specific seroprevalence observed in Hong Kong, while pre-existing immunity in the elderly is not. Overall, our results suggest that stratified immunity in an aged-structured heterogeneous population plays a significant role in determining the shape of influenza epidemics.

## Highlights

• The disease model with stratified immunity improves the accuracy on influenza epidemic reconstruction.
• Antibody boosting in children is greater than adults during influenza outbreak.
• Age-specific mixing pattern and the relative infectivity of children to adults are the key drivers of infection heterogeneity.

## 1 Introduction

Traditional syndromic surveillance for influenza has substantial public health value in characterizing epidemics. For example, by comparing syndromic incidence for one year with previous years, accurate alerts can be issued for possible excessive demands on health services (Ortiz et al., 2009). Epidemic models are commonly used in combination with surveillance data for pandemic prevention, control, forecasting, early characterization of novel strains, and the investigation of drivers of transmissibility of influenza (Heesterbeek et al., 2015). However, less insight can be obtained from these data than might be expected because the relationship between syndromic data and the true infection events can vary from one population to another and from one year to another (e.g., variability in reporting rates), and thus the actual infection number is difficult to estimate.

Serosurveillance of influenza provides a potentially more accurate way to estimate actual numbers of infected cases (Cauchemez et al., 2012, Briand et al., 2011, Lipsitch et al., 2011). Recent studies rely on combining serological test results with syndromic data from traditional surveillance within epidemic models to make inference on epidemiological processes of influenza (Birrell et al., 2011, Dorigatti et al., 2013, Baguelin et al., 2013). For example, Dorigatti et al. (2013) showed that the third wave of pandemic H1N1 infection in the UK could be explained by the increased transmission and short-term age-specific immune waning after jointly fitting the model to influenza-like illness (ILI) incidence and the serological data. However, up to now, these studies treated serological assays as a dichotomous variable, with individuals classified as uninfected and susceptible if their titre is below a certain threshold and infected and immune if their titre is above that threshold. Typically, a titre of 1:40 (dilution ratio) is used as the threshold because this titre was previously estimated to generate about 50% immune protection (referred to as TP50) (Hobson et al., 1972, Coudeville et al., 2010).

However, certain limitations in the use of such threshold data make the further assessment of epidemiological mechanisms more difficult. First, a wide range of antibody boosting (defined as the increase of the antibody titres in response to infections) has been observed in serological studies (Miller et al., 2010), resulting in the underestimation of infection incidence because low titres are ignored (Cauchemez et al., 2012, Wu and Riley, 2014, Wu et al., 2014). Moreover, the protective titre TP50 only provides partial protection against infection and could differ by virus strains and host ages (Black et al., 2012). Together, the incidence is not able to be accurately captured by the depletion of susceptibles within the models using threshold data. Despite the widespread availability of serological data from many recent influenza serosurveillance studies (Achonu et al., 2011, Dudareva et al., 2011, Iwatsuki-Horimoto et al., 2011, Miller et al., 2010, Waalen et al., 2010, McVernon et al., 2010, Reed et al., 2012), current epidemic models have thus far not explicitly represented individual antibody titre levels and its correspondence with immunity (Heesterbeek et al., 2015). Therefore, a more understanding of how serological responses and protection of individuals affect transmission in a heterogeneous mixing population, is a current scientific goal and should facilitate improved predictive models of influenza (Shaman and Karspeck, 2012, Shaman et al., 2013).

Here, we propose a refinement of the concept of the stratified immunity within an epidemic model for influenza transmissions. We explicitly enumerate all possible titres in standard haemagglutination inhibition (HI) assays and map them onto a variable scale of susceptibility. By coupling the epidemic model with serological responses in different age groups, we are able to investigate key biological mechanisms in stratified immunity, such as antibody boosting and protection in greater detail. We are also able to assess the epidemiological factors, among which age-specific prevalence was produced.

## 2 Results

### 2.1 Crude patterns of titre difference

We first compared the overall pattern of antibody titres between the baseline and follow-up measurements in the study. The influenza pandemic started in early May 2009 with the first confirmed case announced on 1st of May. Virologically confirmed incidence of infection reached its maximum during late September. We obtained HI antibody titres from 523 individuals between 4 July 2009 and 28 September 2009 as baseline titres, and HI titres from 465 individuals during the follow-up (between 11 November 2009 and 6 February 2010) (Fig. S1). We defined T1 to be the average of the time of sampling for the baseline study (11 August 2009) and T2 to be the average day of the follow-up (22 December 2009). Changes in the overall distribution of titres indicated that there has been a substantial epidemic during this period. Between T1 and T2, the proportion of the study population without detectable antibodies decreased from 90.1% to 80.0% with the increase in detectable titres being distributed more to the individuals with higher titres (Fig. S2). For example, increases in titres between 80 and 320 accounted for 65.1% of the decrease observed in the proportion with undetectable titre.

### 2.2 Epidemic model with stratified immunity

**Fig. 1:**
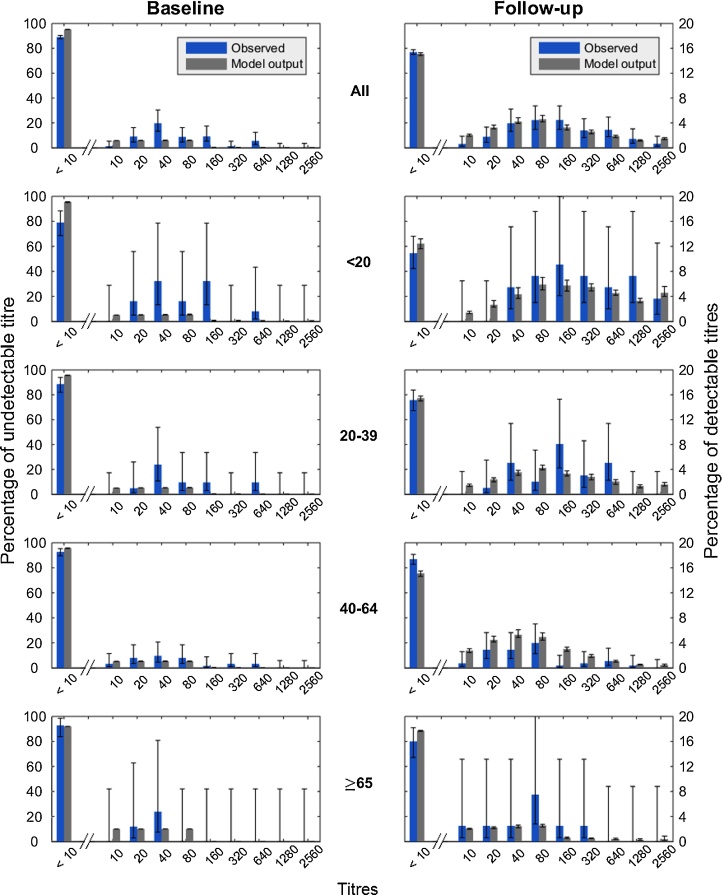
Comparison of titre model fit (gray) and observed (blue) age-stratified data for baseline and follow-up surveys. The top row describes the pattern for the entire population, while the bottom four rows describe patterns for specific age groups. Vertical bars indicate 95% binomial confidence intervals (observed) and 95% region of posterior credibility (model). Left *y*-axis indicates the percentage with undetectable titre. Right *y*-axis indicates percentages in other titre classes. Note left and right *y*-axis are different scales. (For interpretation of the references to colour in this figure legend, the reader is referred to the web version of the article.)

We constructed an age-structured multi-level susceptible-infected-recovered-susceptible (SIRS) epidemic model in which all antibody titres were represented explicitly (referred to here as the model A, the full titre model; see Methods and Fig. S3). Using the titre model with age mixing, we fitted the titre model to the serological data during the pandemic. The fraction of the elderly (≥65 years old, referred as yo) with pre-existing antibodies was set to be twice as large as other persons based on previous studies (Wu et al., 2014, Xu et al., 2011, Riley et al., 2011). Aggregated across age groups, the model was able to reproduce antibody profiles with good accuracy (Fig. 1). Model predicted titres at T1 were slightly lower than certain observed baseline titres but overall were consistent with our observations. At T2, the proportions of each predicted titres fell into or overlapped with the 95% confidence interval of the observed follow-up titres. Children (<20 yo) demonstrated higher antibody titres during follow-up while lower titres were present in the middle-aged adults (40–64 yo) and elderly (≥65 yo). Given the small sample size for the elderly and that pre-existing immunity was not well known, the model fit showed a good agreement with the observed titres across age groups.

### 2.3 Serological responses and clinical protection

We found differences in age-specific antibody boosting following the infection. The average antibody boosting was highest among children, with 62.2 [31.6–128.0] fold increase in titres (*AbB*_1_ = 5.96 [4.98–7.00]; see Table 1). The boosting decreased by age until middle-aged adults to 13.7 [8.17–24.3] fold increase (*AbB*_3_ = 3.78 [3.03–4.60]), which was significantly lower than children (non-overlapping 95% credible intervals). Conversely, the elderly showed higher boosting than middle-aged adults. However, although there was a suggestion that older adults had less protection for a given antibody level, we did not find strong evidence for age-specific correlates of immune protection. The protective titres which were associated with 50% protection (TP50) had overlapping 95% credible intervals and were therefore not significantly different. Among children and adults (≤65 yo), the average protective titres were between 20 and 80, which were consistent with previous studies (Hobson et al., 1972, Coudeville et al., 2010, de Jong et al., 2003). For the elderly, a weaker protective titre was found between 160 and 320, but with a wide credible interval.

### 2.4 Reconstructing epidemic dynamics

**Fig. 2:**
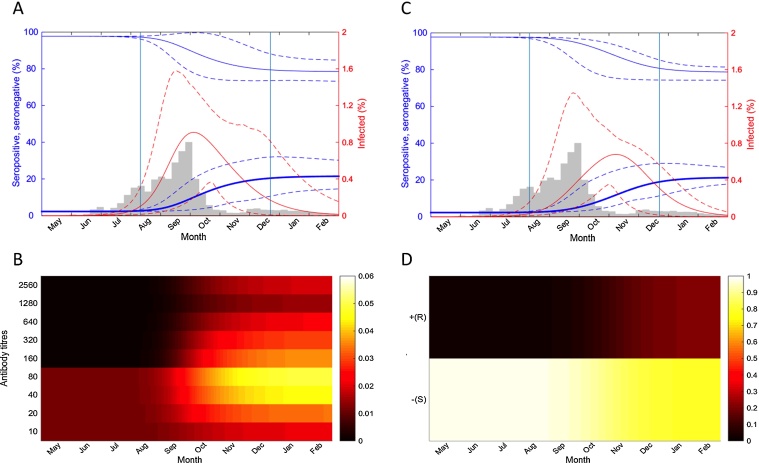
Disease and serological dynamics of the titre model simulation. Dynamics were reconstructed using 400 random samples from the posterior distributions of the parameters. (A) The disease dynamics calculated using the titre model. Bold blue, seropositive individuals, defined as individuals with titres ≥40. Thin blue, seronegative individuals, defined as individuals with titres <40. Solid lines give the posterior mean, while dashed lines give 95% credible intervals. The percentage of the infected individuals is shown in red. Vertical lines indicate average recruiting time T1 and T2 during the periods of baseline and follow-up surveys. Gray bars represent the weekly number of laboratory confirmed cases of 2009 pandemic in Hong Kong. (B) The serological dynamics simulated during the outbreak using the titre model. Darker colour represents a lower proportion and lighter represents a higher proportion of the population with a given antibody titre. (C) The disease dynamics calculated with the threshold model using the classic definition of seropositivity (1:40). Colours are the same as in (A). (D) The serological dynamics simulated during the outbreak using the threshold model. Darker colour represents a lower proportion and the lighter one represents a higher density in the population. Note that the average of the peak time obtained from the posterior sampling is slightly different from the peak of the average incidence as shown in (A) and (C). (For interpretation of the references to colour in this figure legend, the reader is referred to the web version of the article.)

Fitting only serological data, the full titre model was able to reproduce the peak time and the cumulative incidence of the 2009 pandemic in Hong Kong during the initial wave. The average incidence increased rapidly after August and reached the peak on 3 October (Fig. 2A) (note that the peak of the average incidence was slightly different than the average of the peak time on 13 Oct. 2009 [14 Sep. – 29 Nov.]), which was 1–2 weeks later than the observed peak incidence between middle and the end of September using either the hospitalization or laboratory confirmed cases (Riley et al., 2011, Wu et al., 2010). Nevertheless, the few weeks’ delay can be explained by the temporal offset between infection and serological boosting (Miller et al., 2010, Mak et al., 2010, Baguelin et al., 2011). An asymmetric longer tail in the epidemic profile after November was reproduced until the disease faded out in February. The cumulative incidence from 1 May to T2 was 22.3 % [15.5 % −28.1 %] (Table S1), which was consistent with previous estimation (22.5% for 3–59 yo in Wu et al., 2014).

### 2.5 Underestimation of infection incidence from seroprevalence

The predicted seroprevalence, defined as the proportion of the population with titres above or equal to the threshold titre 40, from the full titre model increased from 2.7 % [2.3 − 3.4 %] (T1) to 20.4 % [15.1 − 24.3 %] (T2), which was slightly lower than the baseline titres 8.9 % [6.7 − 11.6 %] but in very good agreement with the follow-up titres 20.6 % [17.2 − 24.5 %] (Table S2 and Fig. S4A). Interestingly, the average increase of seroprevalence (18.1%) from 1 May to T2 was 18.8% less than the average cumulative incidence, indicating that a proportion of nearly 20% of infected individuals was underestimated using seroprevalence, either because individuals had low antibody boosting to titres fell below 40, or were previously seropositive but were still infected or reinfected later due to partial protection (e.g., mostly between titres 40-80, see Fig. 2B).

### 2.6 Model comparison

To compare the full titre model (model A) with the epidemic model that only produced threshold predictions, we performed similar analyses using the susceptible-infected-recovered (SIR) epidemic model (referred to here as the model E, the threshold model; see Methods) fitting to individual seropositivity data aggregated from the same serological titres profile using the threshold titre. Specifically, all titre values below 40 were assigned to be seronegative and all other titre values were assigned to be seropositive. The full titre predictions were also transformed into threshold results. Thus the similarities between model predictions and the individual seropositivity dataset were able to be evaluated for both models. Deviance Information Criterion (DIC) was used to measure the fitness of both models to the seropositivity data (see Methods). Models with smaller DIC were preferred. Generally, differences larger than 5 are substantial. We found that model A was a better explanation of these data than the threshold model E (Δ*DIC* =−11.5).

For both models, the effects of each serological and epidemiological variable were also evaluated. Three alternative restricted titre models, each with age-independent antibody boosting (model B), age-independent antibody protection (model C) and without the relative infectivity (model D), and one alternative threshold model without the relative infectivity (model F) were also compared with model A (see Table 2 for full model descriptions and comparison results). All of the models were able to infer a single peak epidemic between T1 and T2 with the same age mixing and pre-existing seroprevalence. We found that the model C was the best-fit model whereas the model A was comparable to the model C (Δ*DIC* = 2.6) and both models demonstrated similar age-specific antibody boosting (Table S3). Age-dependent antibody boosting (model A versus B) improved the model fit (Δ*DIC* =−7.0) while age-dependent antibody protection (model A versus C) not. This further confirmed the significant differences in age-specific antibody boosting but not among age-specific protection in the full titre model (Table 1). We also demonstrated that the relative infectivity of children (model A versus D) greatly improved the fit of the titre model (Δ*DIC* =−38.5).

### 2.7 Determining age-specific seroprevalence

**Fig. 3:**
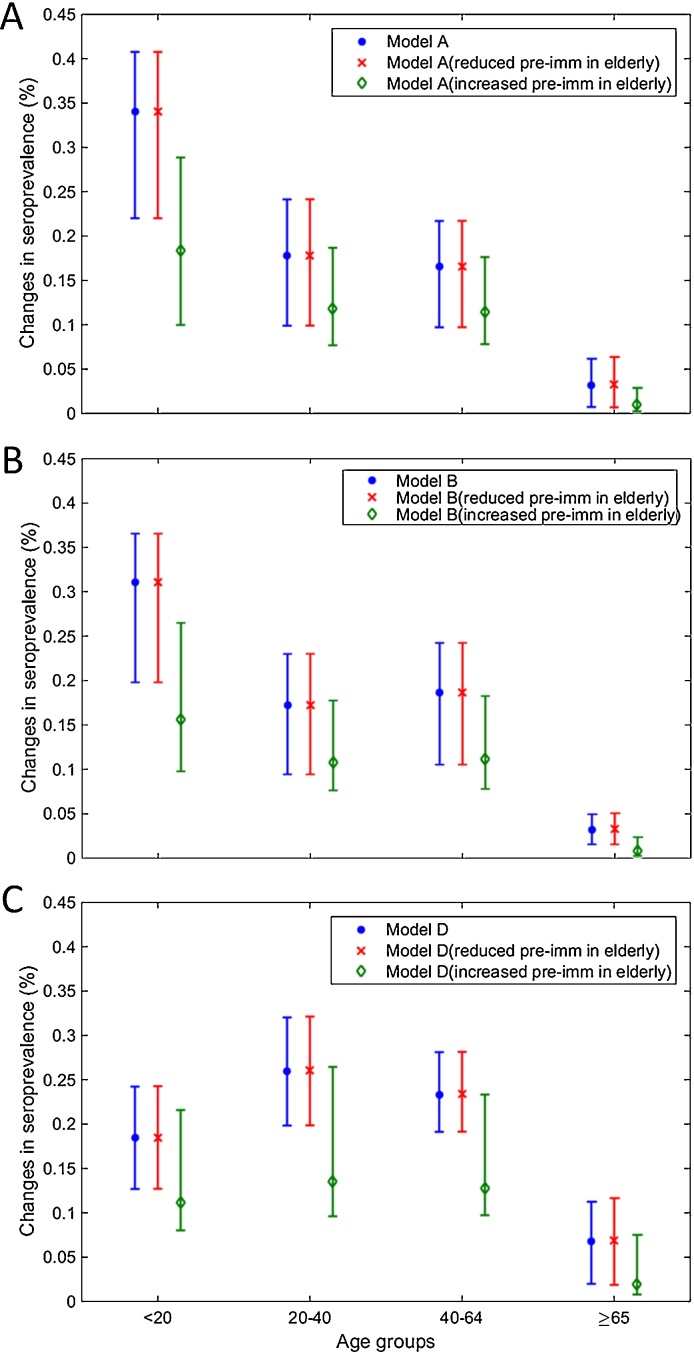
Changes in age-specific seroprevalence under different assumptions on antibody boosting and the relative infectivity of children. (A) Changes in seroprevalence calculated using the titre model with full parameter sets (model A) from the first day of the pandemic until the follow-up recruiting time T2. Blue, the titre model with pre-existing immunity in the elderly set to twice that of other age groups (default setting). Red, the titre model with reduced pre-existing immunity in the elderly and all age groups have the same seroprevalence. Green, the titre model with higher pre-existing immunity in the elderly set to 4 times that of other age groups. (B) Changes of the seroprevalence calculated using the titre model with the same antibody boosting among different age groups (model B). Colours are the same as in (A). (C) Changes of the seroprevalence calculated, using the titre model without the increased relative infectivity of children (model C). Colours are the same as in (A). Note that the changes in seroprevalence in model C were measured from the first day of the pandemic until the follow-up recruiting time T2 plus additional 60 days to adjust for the delay of the peak. Similar patterns of seroprevalence were able to be produced if T2 was used instead of T2 plus 60 days. (For interpretation of the references to colour in this figure legend, the reader is referred to the web version of the article.)

The titre model with age contact mixing allowed us to explore the effects of different factors on determining age-specific infection pattern. As reported from previous studies (Wu et al., 2010, Riley et al., 2011), we found that the largest increase of the estimated seroprevalence was found in children from 2.9 % [2.1 − 4.4 %] to 35.8 % [25.3 − 42.7 %], and the least change was present in the elderly from 4.1 % [4.0 − 4.2 %] to 7.1 % [5.5 − 8.5 %] (Table S2 and Figure S4A). To test whether the high pre-existing immunity determined the low incidence in the elderly, we reproduced the seroprevalence changes, in which elderly had only the same initial antibody protection as other age groups and compared the increase of seroprevalence from 1 May to T2. Despite removing this age-dependent pre-existing immunity, a similar age-specific serological pattern was found, indicating that pre-existing immunity in the elderly was not the critical factor to explain the lower incidence in this age group in Hong Kong (Fig. 3A). When the fraction of elderly with pre-existing antibodies was set to be 4 times that of other age groups, seroprevalence was reduced not only in the elderly but also in all the other groups. We further investigated whether the pattern was determined by age-specific antibody boosting. However, the cumulative incidence in the elderly also demonstrated the lowest proportion of infection in the population (Table S1). We then re-fitted the model, in which antibody boosting was the same for all age groups (model B). A similar age-specific pattern was still shown (Fig. 3B). Interestingly, when we re-fitted the model without relative infectivity of children (model D), the seroprevalence of children largely dropped to below young and middle-aged adults but seroprevalence in adults including the elderly was slightly increased (Fig. 3C). Together, these suggested that relative infectivity of children to adults and age contact mixing, rather than pre-existing immunity in elderly or differences in age-specific antibody boosting, were required to explain the age-specific infection pattern in Hong Kong.

### 2.8 Delay in the peak incidence

**Fig. 4:**
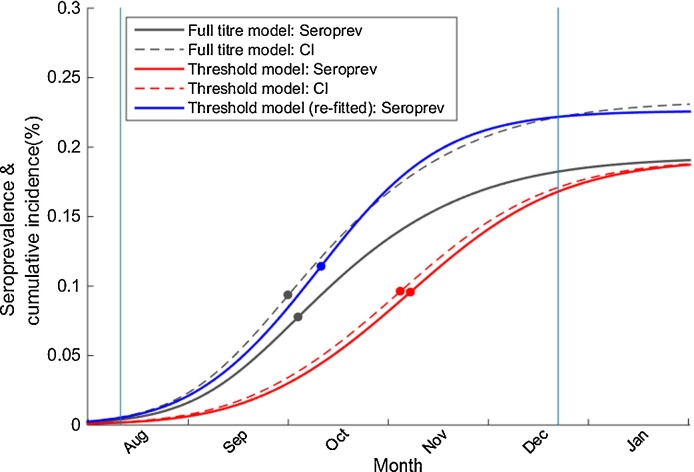
The seroprevalence and cumulative incidence by time reconstructed from the models outputs. Gray, solid and dashed lines represent seroprevalence and cumulative incidence produced by the full titre model (model A). Red, solid and dashed lines represent seroprevalence and cumulative incidence produced by the threshold model (model E). The slight difference between cumulative incidence and the seroprevalence in the threshold model is caused by the assumption that only healthy persons (individuals not in infected status), would participate in the serosurveillance survey. The blue line represents the seroprevalence produced by re-fitting the threshold model to the cumulative incidence generated from the full titre model at T2. The dot represents the time when the maximum slope is achieved, corresponding to the peak of the respected epidemic curve. (For interpretation of the references to colour in this figure legend, the reader is referred to the web version of the article.)

The different epidemic dynamics between the full titre and threshold model highlighted critical challenges with the use of seroprevalence for estimating the cumulative incidence using the classical threshold approaches. Despite the similar seroprevalence between the threshold model E and the observed data (Table S2 and Fig. S4B), the threshold model estimated that the average incidence reached its peak on 6 Nov. (note the peak of the average incidence was slightly different than the average of the peak time: 5 Nov. [23 Sep.–30 Nov.]), which was nearly one and a half months later than the reported incidence peak (Wu et al., 2010, Riley et al., 2011) (Fig. 2C). In the threshold model, because all the infected individuals were assumed to be seropositive after infection (Fig. 2D), the amount of disease incidence not detected by seropositivity, was not identifiable; as a result, the cumulative incidence was 17.1 % [12.9 – 23.0 %] at T2 (Table S1), which was 23.3% lower than the titre model even though both models predicted similar seroprevalence at T2 (Fig. S4). Thus, to produce the reduced final size of incidence, a lower *R*_0_ (or transmission rate) was estimated as 1.19 [1.16–1.25] (Table 1) by the threshold model, resulting in a substantial delayed incidence peak (Fig. 4). We further tested whether peak time could be reconstructed without the delay in threshold model if the cumulative incidence was not underestimated. We re-fitted the predicted seroprevalence to the cumulative incidence generated from the titre model at T2 by changing only *R*_0_. The results demonstrated that if all the incidence was able to be identified in seroprevalence without underestimation, the key epidemiological parameter *R*_0_ would be restored (1.23 [1.20–1.30]) and a similar epidemic curve could be recovered (Fig. 4).

### 2.9 Sensitivity analysis

We tested the robustness of our conclusions to a key sensitivity, by simulating the disease dynamics with different numbers of initial infectious seeds in the population on 1st May. The full titre model was more robust to changes in initial infectious seeds than the threshold model. For the numbers of initial infectious seeds that allowed the titre model to produce the proper peak time similar to the observed reports (e.g., for initial infectious seeds between 10 and 30), the threshold model systematically reproduced a more delayed epidemic peak compared to the titre model (Fig. S5).

### 2.10 Effective reproductive number

**Fig. 5:**
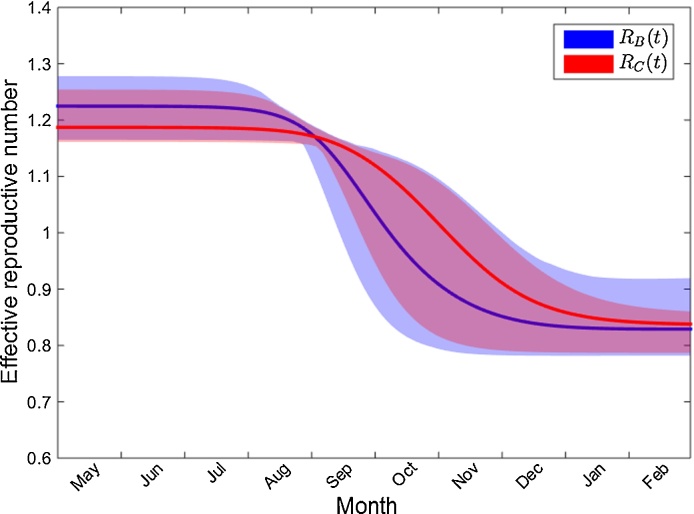
Comparison of the effective reproductive numbers between the titre and threshold models. Blue, the 95% credible interval of the reproductive number *R*_*B*_ estimated from the titre model with age-specific serological parameters. Red, the 95% credible interval of the reproductive number *R*_*C*_ estimated from the threshold model with the same age mixing effect. Bolded lines represent the mean values. (For interpretation of the references to colour in this figure legend, the reader is referred to the web version of the article.)

The titre model allowed us to define the effective reproductive number *R*_*B*_ in the presence of pre-existing antibodies and compared it to *R*_*C*_, defined as the effective reproductive number using the threshold model E. *R*_*B*_ declined slowly from 1.22 [1.16–1.28] in the first four months of the outbreak and dropped rapidly below one during September and October (corresponding to the time of peak incidence); Whereas *R*_*C*_ declined from a lower initial number 1.19 [1.16–1.25] and lasted about 1 month longer before dropping to 1 (Fig. 5), resulting in a reduced cumulative incidence with a delayed incidence peak. Both effective reproductive numbers ended at 0.82, confirming that similar seroprevalences from models were present after the outbreak.

## 3 Discussion

Using time series cross-sectional serosurveillance data, we have demonstrated that explicitly representing stratified immunity improved our ability to explain influenza transmission dynamics. By representing partial protection and the differential boosting of antibodies after infection, we obtained a more accurate epidemic curve in terms of peak time, incidence and the shape of the tail. With partial protection, individuals with weak to medium immunity could still be infected or reinfected, which has been observed (Camacho et al., 2011). It is likely that the longer tail of disease incidence observed in influenza pandemic in Hong Kong after December and in seasonal influenza in the past several years (Russell et al., 2016) may reflect these reinfection dynamics generated by partial protection in the population.

We have refined the concept of the depletion of susceptibles for influenza in epidemic models to incorporate partial immunity and the differential boosting of antibodies, leading to different relationships between seroprevalence and cumulative incidence during the epidemic. A difference has been observed previously (Birrell et al., 2011) between peak incidence inferred with a model fitted to seroprevalence data and syndromic incidence, and was explained by the possible changes of consulting or reporting rates. However, our results suggest that use of threshold immunity within the epidemic model may have underestimated cumulative incidence, resulting in a delay of the inferred epidemic peak, and that the two data sources may be consistent when stratified immunity is taken into account.

The titre model also predicted a larger increase in seroprevalence for children and little increase in elderly persons. However, the low incidence in the elderly could not be explained by higher pre-existing antibodies, as proposed by some other studies (Cauchemez et al., 2009, Ikonen et al., 2010), nor by low antibody boosting. Our results support the importance of age-specific mixing pattern and relative infectivity of children to adults as the key drivers of infection heterogeneity, extending previous work showing that the pre-existing titres and age group mixing alone could not explain the age distribution of infections (Wu et al., 2014). Certain studies have demonstrated that children were associated with higher viral loads or prolonged shedding (Lee et al., 2011, To et al., 2010), which may increase the transmissibility in children.

One of the most important challenges for infectious disease control is to monitor disease transmissibility in real time with statistics such as the effective reproductive number *R*(*t*) (Cowling et al., 2010, WHO Ebola Response and Team, 2014). The titre model structure presented here allows us to define the effective reproductive number in the presence of stratified immunity, *R*_*B*_(*t*). Our analysis suggests that the 2009 pandemic strain had an underlying *R*_*B*_(0) of 1.22 [1.16–1.28] in the presence of the pre-existing or cross-reactive antibodies, which is similar to but slightly lower than *R*_0_ (1.28 [1.23–1.34]) estimated by Wu et al. (2014), when an age-structured transmission model was fitted to both seroprevalence and hospitalization data. In a previous study using laboratory confirmed cases in Hong Kong, the initial effective *R* was estimated around 1.4–1.5 (Cowling et al., 2010) but the number quickly dropped to between 0.9 and 1.3 from July and became lower than 1 after September, which is generally consistent to *R*_*B*_. The difference of *R*_*B*_(*t*) during the initial phase of the epidemic could be due to the absence of school vacations in the model framework presented here.

Our dynamical model with stratified immunity enables us to reduce uncertainty in the number of infected individuals derived from reporting rates; thus potentially providing a framework to improve current predictive modelling of influenza, which essentially relies on the estimates of ILI or relevant influenza cases (Shaman and Karspeck, 2012, Shaman et al., 2013). Planning and implementing intervention strategies such as vaccination, to reduce influenza transmission is currently still a challenge (Lipsitch et al., 2011, Baguelin et al., 2013). The framework here can be extended to evaluate the impact of vaccination on disease transmission through antibody responses. Furthermore, it seems plausible that this general approach can be applied to seasonal influenza viruses and other emerging infectious diseases where partial protection may be important, such as avian influenza, dengue, and Zika viruses, (Duffy et al., 2009, Lanciotti et al., 2008).

## 4 Methods

### 4.1 Serological samples

We analyzed the updated laboratory test results from the baseline and follow-up rounds of the Hong Kong influenza serological survey (Riley et al., 2011). Here, we used HI assay results for the 2009 pandemic strain of H1N1. We obtained the baseline HI titres from 523 individuals (between 4 July 2009 and 28 September 2009) and from 465 individuals recruited during the follow-up period (between 11 November 2009 and 6 February 2010) (Fig. S1). The previously published analyses used only microneutralization assay results for the subset of individuals with the paired samples. We also noted that previous analysis was based only on 4-fold rise or greater in paired samples and thus did not need to exclude individuals who reported vaccination prior to the baseline visit. Here, because it was our objective to make inference on cross-sectional patterns of serology, for our primary analyses, we excluded all individuals who reported any influenza vaccination in the preceding years.

### 4.2 Transmission model

The epidemiological and serological dynamics were simulated based on a disease transmission model with a serological response component. The model is illustrated in Fig. S3A as an extension of the SIRS model, where hosts are classified into discrete immune classes, corresponding to different antibody dilution ratios ranged from <1 :10 (undetectable titre) to 1:10 and from 1:10 to the highest dilution tested 1:2560. To simplify the figure, we exclude age mixing effects in the schema. The probability of susceptible individuals being infected given a contact with an infected person was dependent on their antibody titres. The susceptibility decreased as the individuals’ antibody titre increased (Fig. S3B). Once the infected individual had recovered, the antibody titre was boosted to a higher level according to a truncated Poisson distribution (Fig. S3C) to capture the antibody titre profile as seen in Fig. 1. At the same time, the infected individual became transiently fully protected within 25 days on average by short-lived immunity, possibly mediated by cytotoxic T lymphocytes with other immunological factors (Ferguson et al., 2003, Kaech and Cui, 2012). The individuals later became susceptible again after the transient immunity waned and were assumed to be protected by only antibodies. The epidemiological parameters we used are described in Fig. S3 and Table S4. The variables of the titre model are listed in Table 2. Disease dynamics are described by the following Eqs. (1), (2), (3) with age mixing effects. The model did not take into account age demographics since the duration of the outbreak we considered here was less than one year. Birth and death rates were not included because that the rates of 1/70 per year did not produce marked differences in epidemic curves using the SIR model.

(1) $$\frac{{\mathrm{dS}}_{i}(a)}{\mathrm{dt}}=-S_{i}\cdot \rho_{a}(i)\cdot \lambda (a)+\omega \cdot R_{i}(a)$$

(2) $$\frac{{\mathrm{dI}}_{i}(a)}{\mathrm{dt}}=S_{i}\cdot \rho_{a}(i)\cdot \lambda (a)-\frac{1}{T_{g}}\sum_{j=i+1}^{i_{\max}}I_{i}(a)\cdot g_{\mathrm{ji}}$$

(3) $$\frac{{\mathrm{dR}}_{i}(a)}{\mathrm{dt}}=\frac{1}{T_{g}}\sum_{j=0}^{i-1}I_{j}(a)\cdot g_{\mathrm{ij}}-\omega \cdot R_{i}(a)$$

where *a* represents the age group for each individual, *ρ*_*a*_(*i*) is the disease susceptibility for susceptible individuals in age group *a* in the presence of titre level with index *i* ranging from zero to the maximum index value *i*_*max*_, *λ* is force of infection, *ω* is the waning rate for short-lived transient immunity, *T*_*g*_ is the duration of infection, and *g_ji_* is the probability of immune boosting from titres *i* to *j*. The force of infection on members of age class *a* in a completely naive population (*S*_0_ without antibody protection) is defined as

(4) $$\lambda (a)=\beta \sum_{b=1}^{a_{\max}}\{m_{\mathrm{ab}}\sum_{i=0}^{i_{\max}}f_{b}\cdot I_{i}(b)\}$$

where *m*_*ab*_ is the contact rate from age class *b* to *a*, *f*_*b*=1_ is the relative infectivity of children to adults, and *β* is a scale factor such that the product of *β*, *m*_*ab*_ and *f*_*b*_ becomes the transmission rate in an age-structured naive population. We stratified sera samples into age groups, i.e. children and adolescent (<20 yo; for convenience, we defined this group as children throughout the study), young adults (20–39 yo), mid-age adults (40–64 yo) and elders (≥65 yo). A contact mixing matrix of the four age groups was calculated from a community study in Hong Kong (Fig. S6).

### 4.3 Differential susceptibility

The susceptibility *ρ* is defined as the proportion of individuals that develop disease from infection given a particular titre level. *ρ* is modelled as a two parameters logistic function (Dunning, 2006):

(5) $$\rho (i)=\frac{1}{1+e^{I_{\beta }(i-\mathrm{TP}50)}}$$

where *TP*50 is defined as the titre at which *ρ* will drop 50% from the maximum value (Fig. S3B). *I*_*β*_ determines the shape of the curve, which was assumed to be 2.102 according to a previous study (Coudeville et al., 2010). 1 − *ρ*(*i*) is defined as antibody protection. For individuals with undetectable titre, we assume the maximum susceptibility 100% is present.

### 4.4 Antibody boosting

Antibody boosting *g_ji_* is defined as probability of the increase of serological titre (log scale) from titre index *i* to *j*, which is Poisson distributed but truncated by zero with rate *Abb* (Clifford Cohen, 1960) adapted from a recent serological modelling approach (Kucharski et al., 2015):

(6) $$g_{\mathrm{ji}}=\frac{{\mathrm{Abb}}^{\delta }e^{-\mathrm{Abb}}}{\delta !(1-e^{-\mathrm{Abb}})}$$

where *δ* equals *j* − *i* and the mean of the antibody boosting is derived as *AbB* = $\frac{\mathrm{Abb}}{1-e^{-\mathrm{Abb}}}$.

### 4.5 Transmission model with threshold data

The threshold model is a special case of the titre model, in which the titre index *i* ranged from 0 to only 1, corresponding to individual seropositivity data aggregated from the serological titres based on a threshold titre of 1:40 (dilution ratio). All susceptible individuals (*i* = 0) were complete susceptible to infection. Once an infected individual recovered, the individual became seropositive (*i* = 1) and remained fully protected by antibodies, instead of being partially protected. Susceptibility can also be described by the two parameters logistic function (Eq. (5)), where *I*_*β*_ was near infinity (practically a value larger than 100 was sufficient) and TP50 is a number between 0 and 1. We were able to describe the observed dynamics using an SIR epidemic model.

### 4.6 Parameters estimation

The posterior distributions of the parameters, including the transmission rate scale factor *β*, age dependent antibody boosting *AbB*_*a*_, age dependent immune protection *TP*50_*a*_, and the relative infectivity of children (versus adults) *f*_*a*=1_, were obtained from Metropolis-Hastings in Markov Chain Monte Carlo (MCMC) with 10^6^ steps (Table 1) to guarantee an effective sample size (ESS) of greater than 100 for all parameters. Aside from the variable *f*_1_, prior distributions for all the parameters were set to uniform distributions. The mean relative infectivity of children was set to be 4 with a standard deviation of 0.5 compared to other age groups, based on the assumption that infectivity was linearly correlated with viral load, which was about 4-fold higher in children than other age groups (Lee et al., 2011).

**Table 1:** Parameters estimates from the titre model and the threshold model using MCMC. The minimum effective sample size (ESS) is above 100 for all variables. Burn in was 1000 steps in accordance with the Geweke diagnostic test.

Models	A	E	
*R*_0_	1.22 [1.16–1.28]	1.19 [1.16–1.25]	
*AbB*_1_	5.96 [4.98–7.00]	–	
*AbB*_2_	4.97 [4.02–6.02]	–	
*AbB*_3_	3.78 [3.03–4.60]	–	
*AbB*_4_	4.79 [2.16–7.54]	–	
*TP*50_1_	2.15 [0.61–5.41]	–	
*TP*50_2_	3.40 [0.67–9.13]	–	
*TP*50_3_	2.80 [0.60–9.05]	–	
*TP*50_4_	5.08 [0.77–9.69]	–	
*f*_1_*	5.01 [3.96–5.95]	4.57 [3.63–5.58]	

Note that *R*_0_ is defined in the presence of the initial partial immunity here. *AbB* is defined as the mean of the truncated Poisson distribution. We used uniform priors for all parameters other than *f*_1_.

* For the prior distribution of *f*_1_, we used a Gaussian distribution with mean=4 and standard deviation = 0.5 (see Fig. S7) because a 4-fold increase of viral loads was observed for children (Lee et al., 2011).

The starting day of the pandemic was set to 1st May (the date of the first observed case in the Hong Kong pandemic). We assumed the number of the initial infected individuals was 10, which provided a seed size large enough to cause a major outbreak with only a 10% chance of stochastic extinction when *R*_0_ equals 1.25 (Allen and Lahodny, 2012, Hartfield and Alizon, 2013). The initial antibodies among the 40–80 and 10–20 titre groups were both set to be 2% for all the age groups <65 and 4% in the elderly respectively, representing a higher seroprevalence. Because it has been shown that HI assay titres are generally less sensitive than MN assay (Grund et al., 2011), this gave an approximation of 3.3% pre-pandemic seroprevalence in Hong Kong using MN assays (Wu et al., 2014).

The likelihood of observing cross-sectional serological titres was calculated as

(7) $$L(\theta |e_{t1},e_{t2},\ldots ,e_{\mathrm{tN}})=\prod_{n=1}^{N}f(e_{\mathrm{tn}})$$

where *N* is the total number of the samples, *f*(*e*_*tn*_) is the frequency of observing titre *e* of the individual *n* from the model output with the parameter values *θ* at time *t*. The observation error was included, such that for each individual, the frequency of the titre *e* would be the likelihood of observing titre *e* from all possible true titre distributions *P*(*i*).

(8) $$f(e)=\sum_{i=0}^{i_{\max}}P(i)\cdot P(e|i)$$

We assumed the probability of observational error was uniformly distributed as 0.005 for each titre *i* not equal to *e*. The sum of all observation errors including *i* equal to *e* was 1. The *i*_*max*_ was set to 9 corresponding to a 2560 titre. The above two equations were also applied to the threshold data (individual seropositivity data), aggregated from the recruited serological titre profile using the threshold titre 40, with the maximum titre *i*_*max*_ in Eq. (8) set to 1.

### 4.7 Model comparison

We explored the effect of age-specific antibody boosting, age-specific antibody protection, relative infectivity of children in the titre models and the effect of relative infectivity of children in the threshold models (Table 2). We compared the goodness of fit of the different models using Deviance Information Criterion (DIC) (Spiegelhalter et al., 2002) to individual seropositivity data *y*, aggregated from the recruited serological titre profile using the threshold titre 40. Among each model variant, the log-likelihood of observing seropositive samples with parameters *θ* was calculated using Eqs. (7) and (8) where *i*_*max*_ equals 1. DIC was derived as $\mathrm{DIC}=P_{D}+\bar{D}$ using 400 random samples from the posterior distributions, where deviance *D* is defined as −2 · *log L*(*θ*|*y*), *P*_*D*_ equals $\bar{D}-D_{\bar{\theta }}$, $\bar{D}$ is the expectation of *D*, and $\bar{\theta }$ is the expectation of *θ*.

**Table 2:** The variables specified in the full titre model and the alternative versions of the titre and threshold models.

Models	A	B	C	D	E	F
Basic reproductive number	✓	✓	✓	✓	✓	✓
Age-dependent Ab boosting	✓		✓	✓		
Age-dependent Ab protection	✓	✓		✓		
Relative infectivity of children	✓	✓	✓		✓	

Age-independent Ab boosting		✓				
Age-independent Ab protection			✓			

Δ*DIC*^a^	0	7	−2.6	38.5	11.5	15.4

a Note that Δ*DIC* was calculated as the *DIC* of the alternative model minus the *DIC* of the full titre model (719.7).

### 4.8 Calculating effective reproductive number

The effective reproductive number was calculated using next generation matrix approaches. Following the same notation as in the study by Diekmann et al. (2010), the transmission matrix *T* and the transition ∑ can be obtained. Each element in *T* represents the average newly infected cases in age group *a* and titre index *i* in a unit time transmitted by single infected individual in age group *b* and titre index *l*, which can be calculated as *βM*_*ab*_*f*_*b*_*S*_*i*_(*a*)*ρ*_*a*_(*i*). ∑ represents the transitions between cases in each age and titre group. Since the probability of boosting to all other titres becomes one, each element in ∑ is simply the loss of infected cases due to recovery in a unit time −1/*T_g_* from our model.

### 4.9 Contact mixing

To quantify and describe the tendency of people to mix with others of similar or different ages, an age-specific contact matrix of participants was constructed based on four waves/rounds of recruitment of the longitudinal telephone contact survey (Kwok et al., 2017). Four groups for age of participant were defined as columns (2–19,20–39,40–64,65+) and four groups for age of contacts (0–19,20–39,40–64,65+) were defined as rows, and were calculated based on the number of daily contacts between individuals based on their age group.

## Author's contribution

HY, SR and MB designed the experiments. SR and KOK collected data. HY performed the experiments and analyses. All authors discussed the results. HY, SR and KOK drafted manuscript. EVL and NA revised the manuscript.
