# Supplementary material for: The impact of stratified immunity on the transmission dynamics of influenza
Source: Epidemics. 2017 Sep;20:84–93. doi: 10.1016/j.epidem.2017.03.003 (PMC5628170; doi:10.1016/j.epidem.2017.03.003)
Supplement: Supplementary file 1 [file mmc1.pdf]

Supporting Information

Table S1. Comparison of the cumulative incidence (%) in age groups from both the full titre and threshold model. Cumulative incidence was calculated from the first day of the pandemic until the follow-up recruiting time T2.

Ages	Full titre model A	Threshold model E
All	22.3 [15.5 - 28.1]	17.1 [12.9-23.0]
<20	36.4 [25.1 45.7]	20.8 [11.3 34.6]
20-39	20.6 [14.5 26.3]	11.3 [6.0 19.0]
40-64	22.5 [15.8 28.9]	12.3 [6.5 20.7]
$\geq$ 65	3.9 [2.7 5.0]	2.0 [1.1 3.6]

Table S2. Comparison of the observed seroprevalence and the seroprevalence from both the full titre and the threshold model. The seropravalence (%) among each age group during the baseline and the follow-up rounds are listed.

	Observed		Full titre model A output		Threshold model E output	
Ages	Baseline	Follow-up	Baseline	Follow-up	Baseline	Follow-up
All	8.9 [6.7 11.6]	20.6 [17.2 24.5]	2.7 [2.3 3.4]	20.4 [15.1 24.3]	2.4 [2.3 3.0]	18.9 [14.5 25.5]
<20	17.7 [10.4 29.5]	45.5 [33.7 59.4]	2.9 [2.1 4.4]	35.8 [25.3 42.7]	2.3 [2.1 3.4]	30.0 [22.5 41.4]
20-39	10.5 [6.0 18.0]	23.2 [16.2 32.8]	2.4 [2.1 3]	19.8 [14.4 23.9]	2.2 [2.1 2.6]	17.6 [13.3 23.9]
40-64	5.1 [3.2 8.2]	9.4 [6.5 13.5]	2.3 [2.1 2.9]	18.4 [13.2 22.6]	2.2 [2.1 2.7]	18.4 [14.4 25.8]
≥ 65	4.8 [1.5 16.2]	15.0 [7.3 29.8]	4.1 [4.0 4.2]	7.1 [5.5 8.5]	4.0 [4.0 4.1]	7.0 [6.1 8.3]

Table S3. Parameter estimates from the alternative titre and threshold models using MCMC. The minimum effective sample size (ESS) is above 100 for all variables. Burn in was 1000 steps in accordance with the Geweke diagnostic test.

Models	B	C	D	F
R_0	1.22[1.16-1.28]	1.24[1.18-1.28]	1.15[1.14-1.17]	1.19 [1.16-1.25]
AbB_1	-	5.95[5.04-6.97]	6.11[4.98-7.35]	-
AbB_2	-	4.98[4.05-6.01]	5.03[4.05-6.12]	-
AbB_3	-	3.87[3.16-4.66]	3.78[2.97-4.65]	-
AbB_4	-	4.86[2.39-7.50]	4.64[2.13-7.29]	-
$TP50_1$	2.28[0.60-6.04]	-	5.23[1.00-9.65]	-
$TP50_2$	3.71[0.70-9.26]	-	3.37[0.70-8.86]	-
$TP50_3$	2.15[0.58-7.85]	-	2.96[0.62-9.09]	-
$TP50_4$	5.17[0.83-9.72]	-	5.45[0.89-9.72]	-
f_1	4.96[3.88-5.94]	5.08[4.14-5.93]	-	-
AbB_{1-4}	4.85[4.33-5.40]]	-	-	-
$TP50_{1-4}$	-	1.55[0.57-3.23]	-	-

Table S4. The parameters used in the titre and threshold models.

Parameters	Descriptions	Values
T_g	Infectious period	3.3 (day)
ω	Recovery rate	$1/25$ (day^{-1})
I_β	Protection shape	2.102
N_{tot}	Total population size	$7 \cdot 10^6$ $(person)$
N_a	Proportion of age groups	[17.6 29.7 39.4 13.3] (%)
I_0	Initial seeding	10 $(person)$

Total population size N_{tot} and the proportion of age groups N_a are derived from the Hong Kong population census report ([50]).

References

[50] 2011 Population Census Office, Census Department, and Statistics in Hong Kong,2011. Hong Kong 2011 Population census summary results, Available from:http://www.censtatd.gov.hk/hkstat/sub/sp170.jsp?productCode=B1120055.

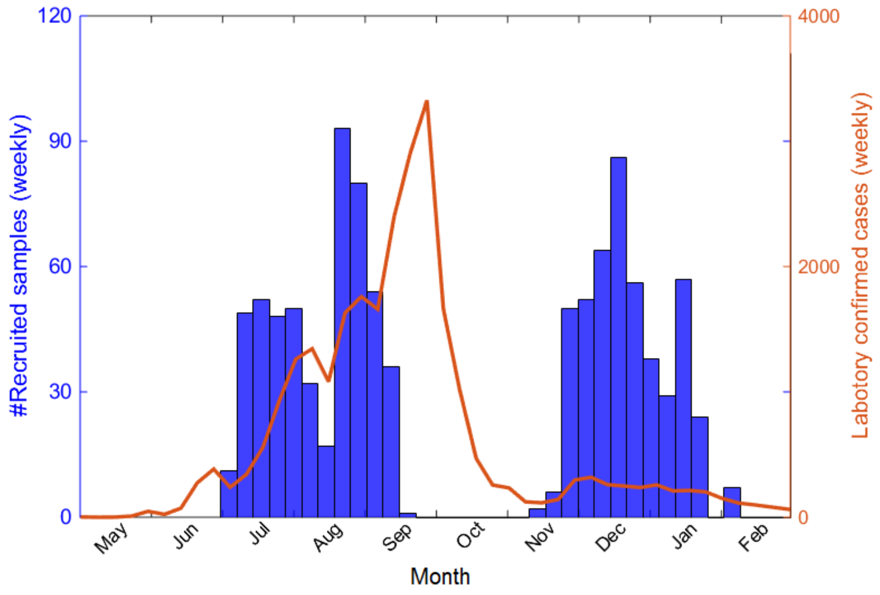

Figure S1. Weekly sampling distribution of baseline and follow-up recruitments during pandemic H1N1 in Hong Kong. We obtained the baseline HI titres from 523 individuals (between 4 July 2009 and 28 September 2009), and from 465 individuals recruited during the follow-up (between 11 November 2009 and 6 February 2010) during early and post pandemic. The laboratory confirmed cases in Hong Kong are plotted for each week ([24]). The peak of the incidence occurred in the end of September.

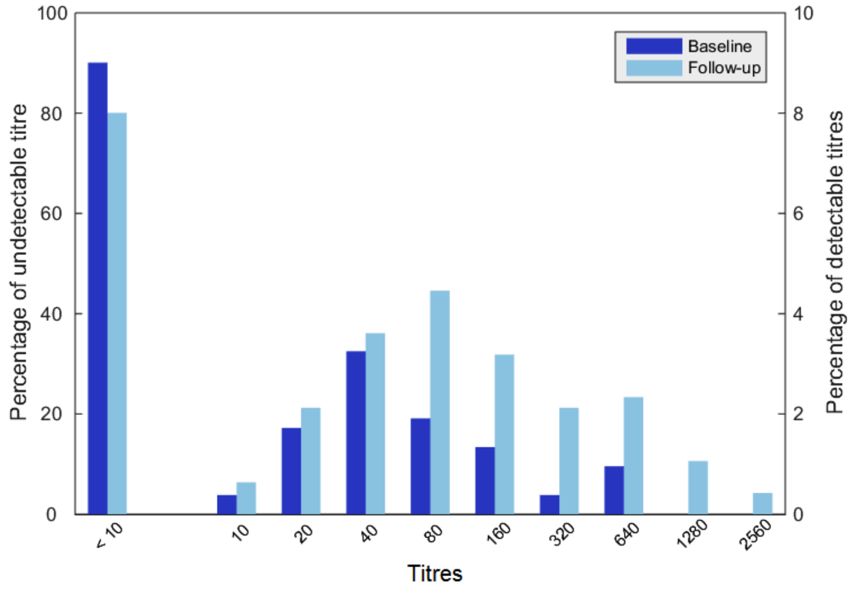

Figure S2 The serological profiles of naive (left y-axis) and immune population (right y-axis) during the baseline and follow-up rounds. Dark blue bars represent the baseline titres and light blue bars represent follow-up titres. Left y-axis indicates percentage with undetectable titre. Right y-axis indicates percentages in other titre classes. Note left and right y-axis are different scale.

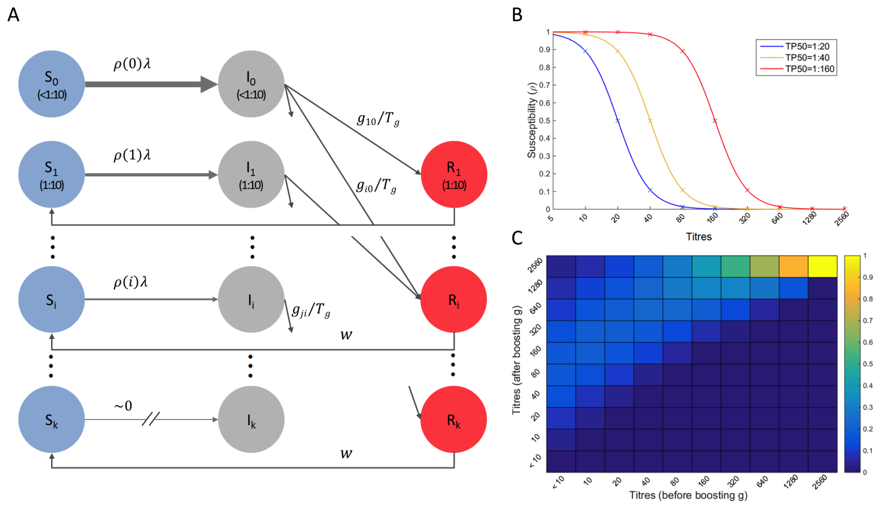

Figure S3. The illustrated schema of the titre model without age mixing effects. (A) In our simulation, the average infectious period T_g was 3.3 days. We assumed that recovered individuals R are fully but temporarily protected within 25 days protected period $1/\omega$ on average. After immunity wanes, recovered individuals become susceptible again and protected by antibody titres (indexed by i) only. Individuals with a lower titre index (i) are more susceptible to infection $\rho(i)$. The force of infection λ is defined in equation (4). After each recovery event, antibody titres i will be boosted to a higher level j with probability g_{ji}. We assumed the maximum index k to be 9, corresponding to a measurable titre of 2560. Any titres larger than this value would be treated as 2560 in our model. The total population size is assumed to be $7 \cdot 10^6$. (B) Susceptibility of individuals to infection given different antibody titres $\rho(i)$ is determined by TP50. 3 $TP50$, 1:20, 1:40, and 1:160 dilutions were used to show susceptibility from higher to lower protection, defined by a two parameters logistic equation as equation (5). (C) The probability distribution of antibody boosting g_{ji} after an infection event. The columns represent the titres i before antibody boosting and the rows represent the titres j after antibody boosting (see equation (6)).

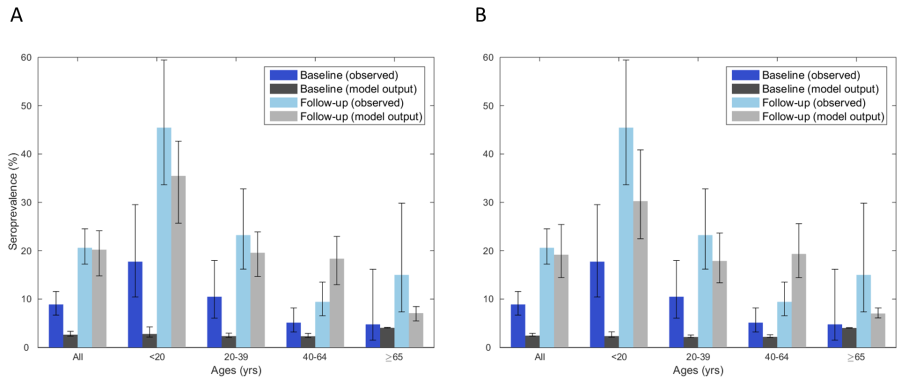

Figure S4. The seroprevalence (percentage of individuals with titres ≥ 40) from the observed sera and the titre model output during baseline and follow-up periods among different age groups. (A) The predicted seroprevalence from the full titre model. Darker blue represents the observed sera at baseline where lighter blue represents the observed sera at the follow-up; Darker gray represents the simulated sera at baseline where lighter gray represents the simulated sera at the follow-up. (B) The predicted seroprevalence from the threshold model. The colours are the same as in (A).

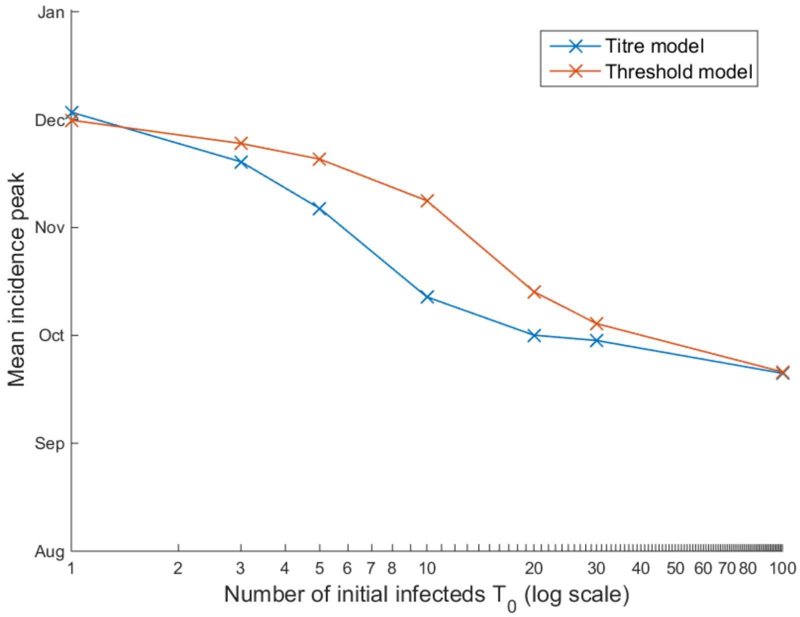

Figure S5. Time of peak incidence among different numbers of initial infecteds T_0. Blue is the estimate of the titre model. Red is the estimate of the threshold model.

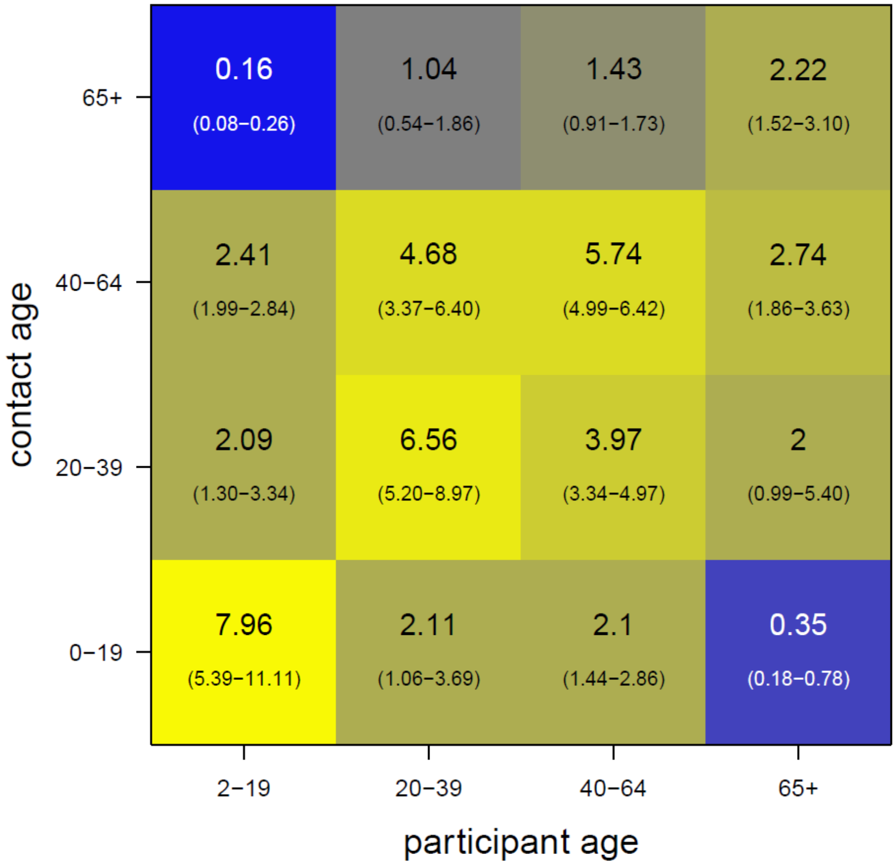

Figure S6. Average daily number of contacts made between age classes. Bluer colours indicate less mixing between age groups than expected by random mixing, and yellower colours indicate more mixing. 95% confidence intervals are shown in the parenthesis, derived from 1,000 re-samples of participant contact diaries. Note that for participants who provided more than one diary, only one diary was randomly drawn in each run of bootstrap

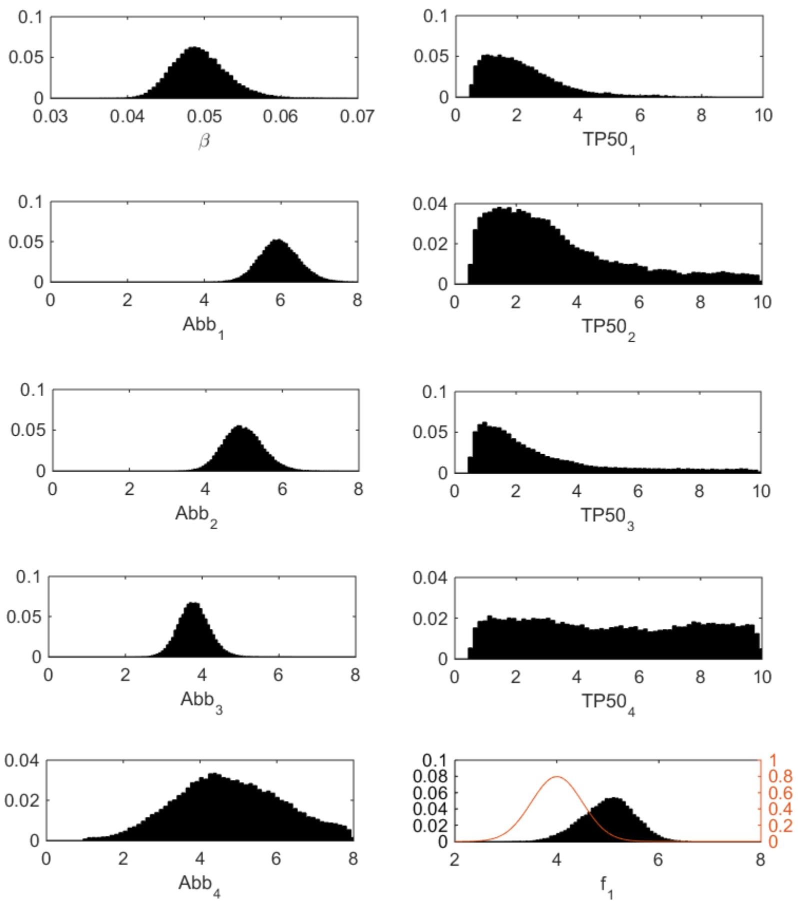

Figure S7. Posterior distributions of the parameters in the full titre model. We use uniform priors for all parameters except f_1. For f_1, we use Gaussian distribution with mean=4 and standard deviation=0.5 because a 4-fold increase of viral loads in pH1N1 was derived for children (0-14 yo) compared to adults ($\geq$ 20 yo) normalised by demographic density in Hong Kong. The Gaussian prior is plotted in red. All 4 $TP50$ parameters show little correlation where a maximum correlation coefficient of 0.19 between $TP50_1$ and $TP50_3$.
